# Development and validation of a prognosis risk score model for neonatal mortality in the Amhara region, Ethiopia. A prospective cohort study

**DOI:** 10.1080/16549716.2024.2392354

**Published:** 2024-08-30

**Authors:** Mengstu Melkamu Asaye, Yohannes Hailu Matebe, Helena Lindgren, Kerstin Erlandsson, Kassahun Alemu Gelaye

**Affiliations:** a Department of Women and Family Health, School of Midwifery, College of Medicine and Health Sciences, University of Gondar, Gondar, Ethiopia; b Department of Pediatrics and Child Health, School of Medicine, College of Medicine and Health Sciences, University of Gondar, Gondar, Ethiopia; c Department of Women’s and Children’s Health, Karolinska Institute, Solna, Sweden; d Department of Health Promotion Science, Sophiahemmet University, Stockholm, Sweden; e Institution for Health and Welfare, Dalarna University, Falun, Sweden; f School of Health and Welfare, Dalarna University, Falun, Sweden; g Department of Epidemiology and Biostatistics, Institute of Public Health, College of Medicine and Health Sciences, University of Gondar, Gondar, Ethiopia

**Keywords:** Neonatal resuscitation, birth asphyxia, newborn, neonatal mortality, clinical decisions, Ethiopia, near miss, prediction, risk score, Sustainable Development Goals

## Abstract

**Background:**

A neonatal mortality prediction score can assist clinicians in making timely clinical decisions to save neonates’ lives by facilitating earlier admissions where needed. It can also help reduce unnecessary admissions.

**Objective:**

The study aimed to develop and validate a prognosis risk score for neonatal mortality within 28 days in public hospitals in the Amhara region, Ethiopia.

**Methods:**

The model was developed using a validated neonatal near miss assessment scale and a prospective cohort of 365 near-miss neonates in six hospitals between July 2021 and January 2022. The model’s accuracy was assessed using the area under the receiver operating characteristics curve, calibration belt, and the optimism statistic. Internal validation was performed using a 500-repeat bootstrapping technique. Decision curve analysis was used to evaluate the model’s clinical utility.

**Results:**

In total, 63 of the 365 neonates died, giving a neonatal mortality rate of 17.3% (95% CI: 13.7–21.5). Six potential predictors were identified and included in the model: anemia during pregnancy, pregnancy-induced hypertension, gestational age less than 37 weeks, birth asphyxia, 5 min Apgar score less than 7, and birth weight less than 2500 g. The model’s AUC was 84.5% (95% CI: 78.8–90.2). The model’s predictive ability while accounting for overfitting via internal validity was 82%. The decision curve analysis showed higher clinical utility performance.

**Conclusion:**

The neonatal mortality predictive score could aid in early detection, clinical decision-making, and, most importantly, timely interventions for high-risk neonates, ultimately saving lives in Ethiopia.

## Paper context

- **Main findings:** This prognosis risk score for neonatal mortality tested in Ethiopia had high performance accuracy and the decision curve analysis showed increased clinical utility performance.
- **Added knowledge:** The tool developed here can aid healthcare providers in identifying high-risk neonates and making timely clinical decisions to save lives.
- **Global health impact for policy and action:** The findings have the potential to be applied in local contexts to identify high-risk neonates and make treatment decisions that could improve child survival rates.

## Background

Severe neonatal morbidity in the first 28 days of life can have both immediate and long-term effects on the neonates’ overall health [1]. Early identification, timely clinical decisions, and accurate care are important for identifying complications in the neonate’s first hours, days, and weeks of life, in order to optimize the neonate physically, psychologically, and socially [2,3].

The neonatal period (the first 28 days of life) is critical for child survival [4]. The estimated average global neonatal mortality rate is 18 per 1000 live births [5], and this is higher in low-income countries [6]. According to the 2019 Ethiopia Mini Demographic and Health Survey, the country’s neonatal mortality rate was 30 per 1000 live births [7]. Ethiopia’s Amhara region has the highest neonatal mortality rate (47 per 1000 live births) [8]. The United Nations Sustainable Development Goals state that all countries, including Ethiopia, should aim to reduce neonatal mortality to 12 deaths per 1000 live births by 2030 [4].

A prognostic mortality risk scores for high-risk neonates might help identify areas for improvement in clinical care. Such a score could help clinicians evaluate their practice. It could also encourage healthcare professionals’ engagement in mother to neonate care, promote safety, security, and comfort in the clinical environment, and increase parental trust in the health care professionals’ competence and knowledge. These are all important elements for neonatal health and survival in the first 28 days of life [9].

Potential predictors of neonatal mortality are the 5 min Apgar score of less than 7 [10,11], birth weight less than 2500 g, birth asphyxia, pregnancy-induced hypertension [12–15], women with HIV positive status, and anemia [16,17]. Early identification of at-risk neonates using medical and socio-demographic variables in low-resource settings is one of the preconditions for reducing neonatal mortality [18]. Practical issues concerning distance, transportation, and timing of delivery, as well as previous experiences, social and cultural norms, may affect the woman's and family’s timely preparation for delivery at a health center or hospital [19]. In clinical practice, the ultimate goal of all diagnostic and therapeutic decisions is to improve prognosis [20]. Clinical prognosis models developed using clinical predictors can help determine the likelihood of a particular patient’s outcome. They can aid in individualized diagnostic and therapeutic decision-making in health care practice [21]. The accuracy of a clinical prognosis model depends on a careful analysis of the relationship between predictors and outcomes, as well as how predictors interact [22].

In the neonatal setting, a clinical prognosis model combines predictors to provide individual predictions regarding neonatal mortality [23]. This can assist in the early identification of high-risk neonates and timely clinical decision-making when the net benefit is greater than treating all or no participants [24,25]. Decisions based on an accurate model can save neonates’ lives by early admission as well as by reducing unnecessary admissions [26]. However, inaccurate clinical decisions can be made as a result of poorly calibrated prediction models [27].

A prognosis risk score model based on clinical variables can assist in evidence generation and early decision-making. To date, no previous studies in Ethiopia have predicted the possibility of neonatal mortality in clinical care. Against this background, the objective of this study was to develop and validate a prognosis risk score for near-miss neonates in six selected public hospitals of Amhara region, Ethiopia.

## Methods

### Study design and setting

A multicenter prospective cohort study was conducted among near-miss neonates in six public health hospitals in the Amhara Regional State, Ethiopia, from July 2021 to February 2022. University of Gondar Comprehensive Specialized Hospital, Debre Tabor General Hospital, Debark Primary Hospital, Gaynt Primary Hospital, Debre Markos Referral Hospital, and Felege Hiwot Comprehensive Specialized Hospital were randomly selected. Every maternity ward has triage, follow-up, second stages, and postnatal units. The new-born wards were divided into sections. Each ward has senior doctors, midwives, and nurses. The average monthly birth rate ranged from 135 to 340 [28]. Each year, approximately 15,000 births occur at these hospitals, with approximately 2450 neonates admitted to neonatal intensive care units. Data on mother-new-born pairs were collected through interviews and reviews of medical records.

### Participant selection

The study included near-miss neonates, who were newborns in labour wards or born in the selected hospitals and admitted to a NICU within 24 h after leaving the labour wards, and who met at least one criteria of the validated neonatal near-miss assessment scale (bradycardia <80 bpm, positive pressure ventilation, intubation for suctioning, inability to suck within 12 h of birth, recurrent seizure) [28,29]. Three hundred and sixty-five (365) near-miss neonates were enrolled in the study. Mobile phone numbers of family or other contacts were recorded for follow-up.

### Ethics

The neonate’s mother provided written consent before data collection began. Mothers of neonates were given written and oral information about the study’s objective and procedures. It was emphasized that participation was entirely voluntary and that non-participation would have no effect on neonatal care or treatment. Informed consent was obtained in writing or with a thumbprint. All data were anonymous and used only for the study’s purpose.

### Variables and outcome measures

The Neonatal Near-Miss Assessment Scale (NNMAS) [28,29] was used to enroll the Near-miss neonates immediately after birth or first day for NICU admission. Outcome: Neonatal death (Yes/No) defined as neonatal death within 7 days postpartum or neonatal death within 28 days of post-partum among enrolled near-miss neonates. Outcomes of admitted near-miss cases were determined through a review of medical records kept by health professionals. Phone calls by data collectors and local health extension workers were used to determine the outcome of discharged near-miss neonates. Three attempts on different days were made to contact either the mother or a relative [30].

Predictor assessment: The variables were developed by reviewing relevant literature [10–17] and experts’ judgement in the field (specific training, maternal and new-born health experts) to identify candidate predictors that could predict neonatal mortality within 28 days among Near-miss Neonates. Pregnancy and newborn-related predictors were collected at the start of the study to predict neonatal mortality. Pregnancy-related predictors were rural residence, maternal age, women with HIV-positive status, iron-folate supplementation, planned pregnancy, pregnancy-induced hypertension, anemia during pregnancy (hemoglobin less than 11 g/dl), inter-birth spacing, antenatal malaria prevention counselling, and fetal movement reduction. New-born related predictors were a 5 min Apgar score, sex of the new-born, birth asphyxia (the failure to initiate and sustain breathing at birth) [31], birth weight, gestational age, and NICU admission.

### Data collection and quality assurance

Training was conducted with nine data collectors and three supervisors in regard to the purpose of the study, data collection and storage, and data collection supervision. A pilot study was conducted to ensure that data collectors and supervisors were qualified to perform and supervise data collection [28]. Following the pilot study, question disarrangement and data incompleteness were identified, and corrective measures were taken. Data were checked for completeness, coded, and entered manually into Epi-Info version 7.1.2. The data were exported to STATA Version 16 for verification, cleaning, and analysis. The data were checked by summarizing and cross-tabulating predictors and outcome variables. All statistical analyses were performed using the Stata Version 16.0 software.

### Data processing and analysis

We used lasso logistic regression for variable selection and regularization to improve prediction accuracy, and the interpretability of findings for short-term prognostic events (28-day mortality) [32]. Model performance was determined by both discrimination (the ability of predictors to distinguish between groups; dead and surviving neonates, measured using the receiver operating characteristics curve or C-statistics) and calibration (measures the degree of agreement between the predicted and observed values) [33]. For missing data, Little’s test was performed and the result was *p* = 0.341, indicating that the missing data was completely at random. Imputation was used to address missing data. The overall death rate was estimated with 95% confidence intervals. Categorical variables were reported using frequency and proportions. There were three phases of model development and analysis as explained below.

### Phase 1: model development

In this phase, a data set containing 20 independent variables was used to develop risk prediction for death. All categorical variables were tested for multicollinearity using the variance inflation factor (VIF). There was no multicollinearity. A logistic regression model was used to conduct bivariate analysis for each individual predictor and the outcome (28-day status). In order to develop the most parsimonious model, 10 prognostic predictors with a P-value < 0.2 were included in the lasso regression for potential predictor selection. The Hosmer–Lemshow test was used to evaluate the calibration performance of the final model’s goodness fit. Calibration in the large and observed/expected (O/E) ratio was estimated. To estimate discrimination performance, the receiver operating characteristics curve (ROC) analysis was used (concordance-statistic). The scaled Brier score for overall performance was also determined.

### Phase 2: internal validation

Internal validation is an important part of predictive modelling. It evaluates the reproducibility of a developed prediction model for the original sample and prevents over-interpretation of the current data. Our prediction model was developed in the development cohort (*n* = 365), and a 500 bootstrap replication sample was generated by sampling ‘n’ individuals with a replacement from the original sample of neonates. Bootstrapping was used for resampling because it is the most appealing method for obtaining stable optimism-corrected estimates. ‘Optimism’ describes the risk of obtaining misleading measures of predictive accuracy, mostly due to overfitting. Internal validation was used to provide optimism-corrected performance statistics that can mitigate this effect [34]. The development phase’s final reduced model was built, and parameters (predicted probability and c-statistic) were estimated. The optimism and the optimism-corrected of model discrimination were calculated using Corig–Cboot and Corig–Do, respectively. The Somer’s d correlation statistic was used to assess correlations between observed and predicted values (Dboot). The model calibration was evaluated using Dorig-Dboot, where Dorig was the Somer’s d correlation obtained from the derived data. The difference gave optimism (Do), and its value close to zero indicated that the calibration was optimistic. The optimism-corrected model was calculated using Dorig–Do to adjust the developed model for over fitting [20]. P-values less than 0.05 were considered statistically significant.

### Phase 3: decision curve analysis and risk scores

In the decision curve analysis, admission to neonatal intensive care units could represent treatment for every neonate. The developed model had a significant clinical benefit in terms of early identification of high-risk neonates, timely NICU admission, and the right treatment [24,25].

The coefficients of the variables included in the final model were used to generate risk scores. The coefficients were converted to a rounded number by dividing by the lowest coefficient. Total scores were calculated by adding the coefficients of each significant variable.

## Results

The neonatal mortality rate at 28-days was 17.3% (95% CI: 13.7–21.5). Out of 365 neonates, 304 (83.3%) were admitted to the NICU. Of the neonates who died, 8 (19.5%) were to mothers with HIV-positive status, and 43 (15.6%) to mothers with an inter-birth spacing of less than 24 months. Of the neonates who died, 16 (32.0%) were born to mothers who did not take iron-foliate, 6 (13.0%) to mothers who did not have a planned pregnancy, 25 (31.2%) to mothers who had pregnancy-induced hypertension, and 10 (31.2%) to mothers who had anemia during pregnancy (Table 1).

**Table 1.** Baseline characteristics of neonatal mortality rate among near-miss neonates in public hospitals of Amhara regional state, Ethiopia, 2021 (*n* = 365).

Variables		28-days neonatal status			
		Died (%)		Survived (%)	
Maternal residence					
Rural		23 (21.3)		85 (78.7)	
Urban		40 (15.6)		217 (84.4)	
Maternal age					
<20		5 (19.2)		21 (80.8)	
21–35		50 (16.8)		247 (83.2)	
>35		8 (25.0)		24 (75.0)	
Maternal HIV status					
Positive		8 (19.5)		33 (80.5)	
Negative		55 (17.3)		263 (82.7)	
Iron-foliate supplementation					
No		47 (14.9)		268 (85.1)	
Yes		16 (32.0)		34 (68.0)	
Planned pregnancy					
No		6 (13.0)		40 (87.0)	
Yes		75 (22.3)		262 (77.7)	
Pregnancy-induced hypertension					
No		38 (13.3)		247 (86.7)	
Yes		25 (31.2)		55 (68.8)	
Anemia during pregnancy					
No		51 (15.4)		280 (84.6)	
Yes		10 (31.2)		22 (68.8)	
Inter-birth interval					
≥24 months		20 (16.7)		100 (83.3)	
<24 months		43 (15.6)		202 (84.4)	
Malaria prevention counseling during pregnancy					
No		34 (23.4)		111 (76.6)	
Yes		29 (13.2)		191 (86.8)	
Fetal movement reduction					
No		55 (16.1)		286 (83.9)	
Yes		8 (33.3)		16 (66.7)	
5 minutes Apgar score					
≥7		32 (12.9)		217 (87.1)	
<7		31 (26.7)		85 (73.3)	
Sex of the newborn					
Male		37 (16.6)		186 (83.4)	
Female		26 (18.3)		116 (81.7)	
Birth asphyxia					
No		42 (13.5)		269 (86.5)	
Yes		21 (25.0)		33 (75.0)	
Birth weight					
≥2500		17 (8.3)		188 (91.7)	
<2500		46 (28.8)		114 (71.2)	
Gestational age					
≥37 weeks		20 (7.8)		238 (92.2)	
<37 weeks		43 (40.2)		64 (59.8)	
NICU admission					
No		6 (9.8)		55 (90.2)	
Yes		57 (18.7)		247 (81.3)	

### Model development

Bivariate analysis during model development revealed that 10 predictors were associated with the risk of neonatal death. Maternal residence, lack of iron-folate supplementation during pregnancy, pregnancy-induced hypertension, malaria prevention counseling during pregnancy, anemia during pregnancy, fetal movement reduction, gestational age, birth asphyxia, 5 min Apgar score, and birth weight were considered predictors when P-values< 0.2 (Table 1).

Six potential predictors were selected using the Least Absolute Shrinkage and Selection Operator (LASSO) with logistic regression. The final multivariable regression analysis retained six predictors. The predictors were anemia during pregnancy (AOR = 4.06; 95% CI: 1.29–12.75), pregnancy-induced hypertension (AOR = 2.10; 95% CI: 1.04–4.24), gestational age less than 37 weeks (AOR = 2.76; 95% CI: 1.33–5.76), birth asphyxia (AOR = 5.07; 95% CI: 1.05-10.69), 5 min Apgar score less than 7 (AOR = 2.44; 95% CI: 1.30-4.61), and birth weight less than 2500 g (AOR = 2.66; 95% CI: 1.24-5.68) (Table 2).

**Table 2.** Each predictor’s coefficients and risk scores in the model for predicting neonatal mortality among near-miss neonates in public hospitals of Amhara regional state, Ethiopia, 2021 (*n* = 365).

Predictors	Multivariable analysis			Simplified risk score
	AOR (95% CI)	P-value	ß (95% CI)	
Anemia during pregnancy (yes)	4.06 (1.29–12.75)	0.016	1.402 (0.258–2.545)	2
Pregnancy-induced hypertension (yes)	2.10 (1.04–4.24)	0.038	0.712 (0.042–1.445)	1
Pregnancy malaria prevention counseling (no)	0.60 (0.32–1.12)	0.108*	0.510 (−0.112-1.133)	
Gestational age (GA)<37 weeks	2.76 (1.33–5.76)	<0.01	1.017 (0.282–1.751)	1.5
Birth asphyxia (yes)	5.07 (1.04–10.69)	<0.01	1.623 (0.876–1.751)	2.5
5 minutes Apgar score (<7)	2.44 (1.30–4.61)	<0.01	0.886 (0.241–1.530)	1.5
Birth weight (BW)<2500)	2.66 (1.24–5.68)	<0.01	0.977 (0.216–1.737)	1.5

ß-coefficients. *Variable not significant in the multivariable analysis. Simplified risk score: the coefficients of predictors included in the final model divided by the smallest (0.712). Pregnancy-Induced Hypertension (PIH): new onset of hypertension that arose after 20 weeks of pregnancy and elevated blood pressure (systolic ≥140 or diastolic ≥90mmHg).

Linear predictors for estimated risk of neonatal mortality = 1/(1+exp–(−3.36+1.402*anemia (<11)+0.712*PIH+1.017*GA(<37)+1.623*birth–asphyxia+0.977*BW(<2500)+0.886*5^th^ minute Apgar score (<7)).

**Figure 1.**
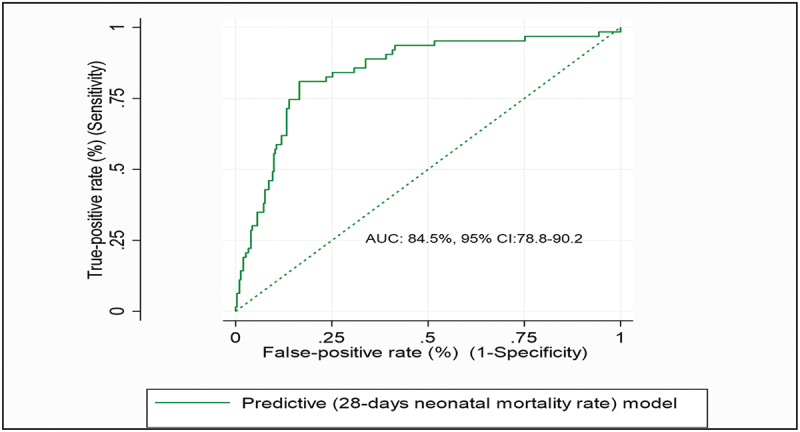
ROC curve of a reduced prognostic model based on six variables to predict the 28-day risk of mortality among near-miss neonates in public hospitals of Amhara regional state, Northwest Ethiopia, 2021 (*n*=365).

The final model had an area under the receiver operating characteristics curve (AUC) of 84.5% (95% CI: 78.8–90.2) (Figure 1). The coefficients’ estimated risk cut-off point was 0.2965, with sensitivity of 73%, specificity of 86%, correctly classified score of 86%, positive likelihood ratio of 5.250, and negative likelihood ratio of 0.313 ROC.

**Figure 2.**
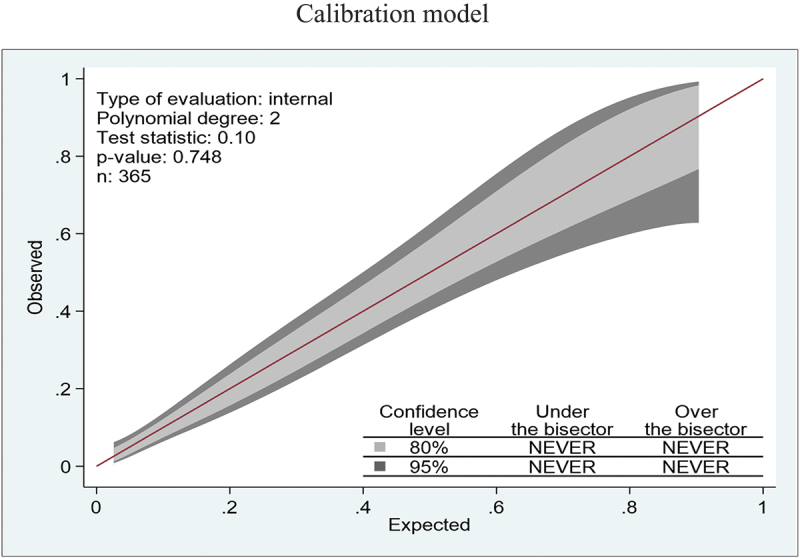
Calibration plot of a reduced prognostic model based on six predictors to predict the 28-day risk of mortality among near-miss neonates in public hospitals of Amhara regional state, Northwest Ethiopia, 2021 (*n*=365).

The P-value of the calibration test was 0.748, indicating that the model was well fit (Figure 2). The calibration in the large (CITL = 0.000, 95% CI:-0.311–0.311), observed/expected ratio (O/E = 1.000), and calibration slope (CS = 1.000, 95% CI: 0.740–1.260) indicated that the model performance parameters were well fit and ideal. The overall brier scaled performance was 22.7% (Figure 2 and Table S1).

### Internal validation

The internal validation of 500–replication bootstrap model performance of the scaled Brier score and C-statistic were 18.8% and (AUC = 0.830; 95% CI: 0.773–0.877), respectively. Its calibration in the large (CITL = 0.015, 95% CI: 0.308–0.341), observed/expected ratio (O/E = 0.991; 95% CI: 0.798–1.191) and calibration slope (CS = 0.923, 95% CI: 0.697–1.203) indicated that the model performance parameters were close to actual.

The estimated optimism and optimism-corrected C-index values of 0.014 and 0.820, respectively, indicated good discrimination. The optimism estimates for the observed-to-expected ratio, calibration in the large, and calibration slope were 0.009, −0.015, and 0.077, respectively. The optimism-corrected calibration of observed-to-expected ratio, calibration in the large, and calibration slope were 0.991, 0.015, and 0.923, respectively. The overall optimism of shrinkage factors was 0.002, indicating there was no over fitting (Table S1).

### Decision curve analysis

**Figure 3.**
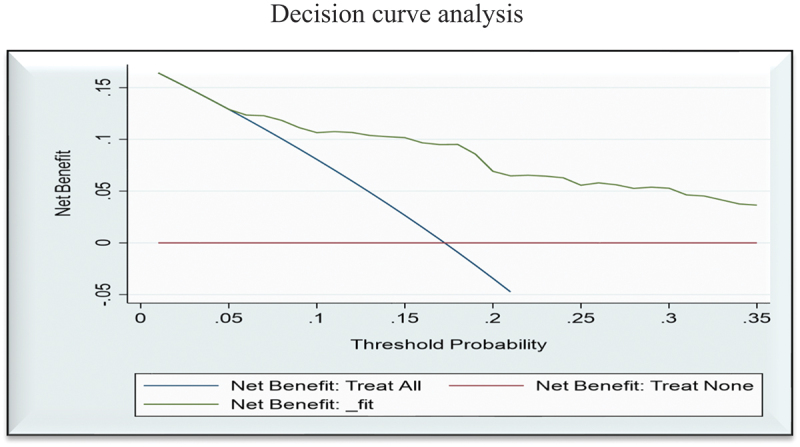
Decision curve plots the model’s net benefit against the threshold probability for clinical utilization of high-risk neonates in public hospitals of Amhara regional state, Northwest Ethiopia, 2021 (*n*=365).

The ‘all’ line in the decision curve plots in Figure 3 denotes the net benefit of treating all neonates, whereas the ‘none’ line denotes the net benefit of treating none of the neonates. Admission to NICU could represent treatment for all. The ‘fit’ line in the decision curve analysis represented the new developed model using multivariable regression coefficients. The ‘fit’ model was the better option for the range of threshold probabilities from 5% onwards. Across the entire range of threshold probabilities, the model had the highest net benefit (higher line than both treating all and no neonates).

### Risk scores

We developed a simplified risk score model to show how it might be used in clinical practice by rounding all regression coefficients. Neonates with a risk score 7 had 48.3% risk of dying. The mortality risk was calculated for each individual risk score, and as the risk score increases, so does the mortality risk. The risk score had an AUC of 82.7 (95% CI: 76.0–88.6). The Youden index was used to classify neonates into low (scores: 0–5) and high (6–10) mortality risks. In neonates with low and high risk of death scores, the mean observed probability of dying was 6.9% (19/275) and 48.9% (44/90), respectively. We estimated the cut-off value of 6, sensitivity as 68.3%, specificity 84.4%, positive likelihood ratio 4.378, negative likelihood ratio 0.376, positive predictive value 47.8%, negative predictive value of 92.7%, and accuracy 81.6% (Table S2).

## Discussion

This study resulted in a novel version of the Prognostic Risk of Neonatal Mortality among near-miss neonates enrolled using the NNMAS [28,29]. The study focused on the provision of care aspects of the WHO quality of care framework [35], and the new evidence informed framework [36]. Local context studies are strongly recommended by WHO for informing policy on how to provide timely quality of care and to improve neonatal survival [37]. The results of this study can inform clinical decision-making to increase child survival rates [38].

The study showed that anemia during pregnancy, pregnancy-induced hypertension, gestational age less than 37 weeks, birth asphyxia, Apgar score less than 7 at 5 min, and birth weight less than 2500 g all predicted neonatal mortality within 28 days of life. A systematic review study [39] confirms these are the clinical/medical causes of neonatal near miss and death for neonates during the first 28 days of life. The findings will be used to assess the clinical conditions of neonates, including decision-making and aspects of nurses’, doctors’, midwives’, and parents’ care and support for vulnerable and sick neonates. We believe that quality improvement interventions in clinical care can reduce neonatal morbidity and mortality rates. A novel version of the Prognosis Risk Score of Neonatal Mortality can be used safely for quality improvement and clinical decision-making purposes. We suggest that WHO re-activate the development of a global NNMAS based on this novel version of prognosis risk score of neonatal mortality and other previously developed instruments [40].

To evaluate the performance of our binary classifier and identify the cut-off to maximize classification accuracy, we used the area under the receiver operating characteristics curve (AUC). The model had an AUC of 84.5%. A value of more than 80% is considered very good discrimination performance [41]. Because of its high performance, the model can assist health care providers in accurately identifying, categorizing, and making timely clinical decisions for risky neonates [42].

The rate of neonatal mortality was 17.3%. To meet the agenda 2030 target of less than 12 per 1,000 live births in all countries, health authorities must develop ways to reduce neonatal mortality [4]. Our study demonstrates how discrimination, calibration, and decision curve analysis measures can be used to indicate the performance of a prognosis model. The prognostic neonatal mortality risk score is more useful in routine clinical practice, and its performance measurements are accurate.

Anemia and pregnancy-induced hypertension are associated with worse neonatal survival rates. There are other newborn-related predictors that can be utilized by healthcare providers to identify and prioritize high-risk newborns and take timely actions to save their lives [12]. Evaluating the quality of care in a fair and reliable manner has always been a critical but difficult task [35]. The multipurpose prognostic scoring system for clinical practice developed here could lead to a paradigm change in which neonatal mortality risk is identified and forecast early using maternal and newborn-related predictors prior to laboratory tests.

Calibration is a performance measure that simplifies the level of agreement between observed and predicted neonatal mortality ratios. It provides information about the calibration of binary outcome models [43]. It compares predicted and observed risks within subgroups of participants and provides information on calibration accuracy [44]. Our model’s performance characteristic was perfectly calibrated at *p*-0.748 and was attached to the belt that encircled the bisector and the 45–degree lines. The P-value of the miscalibration belt, on the other hand, could be significant and deviate from the bisector [45,46]. As a result of poorly calibrated prediction models, inaccuracies and dangerous clinical decisions can be made [27]. Calibration outputs are critical for clinical decision tools. The predicted probabilities have practical importance and are of primary concern to individual patients [47].

Our study found that the prognostic neonatal mortality risk score had high performance accuracy for early identification and timely clinical decision-making. According to WHO, neonates can survive and thrive as productive members of the society in the future if easily accessible and context-based approaches are implemented early [37].

Internal validation of the study’s finding showed near-perfect values in all dimensions [48,49]. The method evaluates the reproducibility of the developed prediction model on the original sample and prevents over-interpretation of the current data. It provides more realistic estimates of the capacity of the model to predict the probability of neonatal mortality. This implies that, using this risk score in clinical practice would be more beneficial for timely admission and for avoiding delays in neonatal interventions. All the results were substantially identical to the original values in the developed model [20,50]. This could be due to the careful candidate predictors’ selection and the application of the lasso regression predictor selection approach [51].

The clinical net benefit analysis found that a better result for clinical decision-making could be advantageous across a wide range of acceptable threshold probabilities. High-risk neonates identified by the score may benefit from a prioritized bundle of labour wards and NICU interventions. The Youden index was used to categorize neonates as low and high risk of death, with high performance and accuracy measures. It suggested that the model had clinical implications. The predictive neonatal mortality risk score model can assist midwives and nurses in labour and NICU wards in low-resource settings such as those in Ethiopia.

A limitation is that the study only included near-miss neonates. This may reduce the prognosis risk score model’s applicability to other neonates. Another possible limitation is that we did not validate the model using a separate dataset because the study had a small sample size. Clinical judgment by experts, lasso regression for variable selection, and internal validation techniques were used to address this issue. We found very small optimism coefficients and large corrected-optimism coefficients, indicating a lack of overestimation biases. This implies that the predictive capability of the model is stable.

## Conclusion

The neonatal mortality predictive score could help in early detection, clinical decision-making, and, most importantly, timely interventions for high-risk neonates, thereby saving lives. Early identification of high-risk neonates can limit avoidable admissions and reduce burdens on costs and other resources. These findings have policy implications for Ethiopia and elsewhere.

## Data Availability

The data sets used and/or analyzed during the current study are available from the corresponding author on reasonable request.

## Abbreviations

AOR = Adjusted Odds Ratio; AUC = Area Under the receiver operating characteristics curve; CI = Confidence Interval; CITL = Calibration In The Large; COR = Crude Odds Ratio; CS = Calibration Slope; LASSO = Least Absolute Shrinkage and Selection Operator; NICU = Neonatal Intensive Care units; NNMAS = Neonatal Near Miss Assessment Scale; VIF = Variance Inflation Factor
